# Flagellar beating forces of human spermatozoa with different motility behaviors

**DOI:** 10.1186/s12958-024-01197-8

**Published:** 2024-03-06

**Authors:** Cristina Tufoni, Alice Battistella, Stefania Luppi, Rita Boscolo, Giuseppe Ricci, Marco Lazzarino, Laura Andolfi

**Affiliations:** 1 University of Trieste, Trieste, 34100 Italy; 2 CNR-Istituto Officina dei Materiali (IOM), SS 14 km 163.5 Area Science Park Basovizza, Trieste, 34149 Italy; 3 Present Address: Institute for Maternal and Child Health - IRCCS “Burlo Garofolo”, Trieste, Italy; 4 Department of Medicine, Surgery and Health Sciences, University of Trieste, Trieste, Italy; 5 Present Address: Max Planck Institute for the Science of Light & Max-Planck-Zentrum für Physik und Medizin, Erlangen, Germany

**Keywords:** Human spermatozoa, Single-cell motility analysis, Flagellar beating forces, FluidFM, AFM

## Abstract

**Background:**

One of the causes of male infertility is associated with altered spermatozoa motility. These sperm features are frequently analyzed by image-based approaches, which, despite allowing the acquisition of crucial parameters to assess sperm motility, they are unable to provide details regarding the flagellar beating forces, which have been neglected until now.

**Results:**

In this work we exploit Fluidic Force Microscopy to investigate and quantify the forces associated with the flagellar beating frequencies of human spermatozoa. The analysis is performed on two groups divided according to the progressive motility of semen samples, as identified by standard clinical protocols. In the first group, 100% of the spermatozoa swim linearly (100% progressive motility), while, in the other, spermatozoa show both linear and circular motility (identified as 80 − 20% progressive motility). Significant differences in flagellar beating forces between spermatozoa from semen sample with different progressive motility are observed. Particularly, linear motile spermatozoa exhibit forces higher than those with a circular movement.

**Conclusions:**

This research can increase our understanding of sperm motility and the role of mechanics in fertilization, which could help us unveil some of the causes of idiopathic male infertility.

**Supplementary Information:**

The online version contains supplementary material available at 10.1186/s12958-024-01197-8.

## Introduction

Mammalian spermatozoa are cells composed of head containing the genetic materials, a neck rich of mitochondria and a flagellum that propels cell through the female tract. A series of biochemical and physiological changes enable them to reach and fertilize the oocyte. Sperm capacitation and hyperactivation are two closely related processes, which occur in spermatozoa during their journey through the female reproductive tract, and they are both critical for successful fertilization [1]. Sperm capacitation is a process in which spermatozoa acquire the ability to fertilize an egg. During capacitation, the spermatozoa are exposed to specific factors in the female reproductive tract that promote their maturation and functional competence, such as removal of inhibitory molecules, changes in ion concentration, and changes in membrane fluidity [2, 3]. In this process the spermatozoa change also their motility pattern to hyperactivated motility. Hyperactivated spermatozoa exhibit a more vigorous and asymmetrical flagellar movement, which allows them to swim more effectively through the female reproductive tract and towards the egg [4]. Hyperactivation is also accompanied by a decrease of flagellar beating frequency, which helps to conserve energy for the journey to the egg. and increase the amplitude and asymmetry of their flagellar waveform, while decreasing their basal beating frequency [5, 6]. Both processes are necessary for successful fertilization, and they are tightly regulated by a complex interplay of cellular and environmental factors. Male infertility is usually diagnosed on the base of several spermatozoa descriptive parameters which include: “oligozoospermia” (reduced sperm density), “teratozoospermia” (reduced percentage of sperm with normal morphology) and “asthenozoospermia” (reduced sperm motility). Asthenozoospermia is a complex and multifactorial condition that can result from a variety of defects in the structure and function of the sperm cell. For example, abnormalities in the axoneme, such as missing or damaged microtubules, can prevent the flagellum from generating the proper waveform. Similarly, defects in the outer membrane can interfere with the flagellum’s movement and cause reduced motility [7].

By using microscopic methods different types of motilities such as progressive, non-progressive or its absence are considered when evaluating sperm behavior [8]. A highly automated computer-assisted semen analysis (CASA) has been developed to analyse sperm concentration, morphology, and motility [9]. CASA can evaluate a variety of motility parameters including: speed, curvilinear velocity, and straight-line velocity. Further information about sperm flagellar activity have been provided by high-speed 2D and 3D optical approaches to reconstruct the flagellar motion in 3D [10]. However, image-based approaches cannot quantify some features of spermatozoa motion, such as, above all, the forces associated with flagellar beating. The forces generated by the motile human spermatozoa are an aspect poorly considered.

There is a growing understanding that forces and mechanical stimuli play a significant, if not essential, role in different biological processes as cell differentiation, embryological development, cancer progression, and immune [11–15]. Likewise, the cellular mechanical characteristics of different cell types and gametes have been demonstrated to be related to their maturation stage and physiological status [16–18]. The fertilization involves a series of consecutive steps that start with the sperm and oocyte membranes recognition, sperm cell penetration of Zona Pellucida (ZP) and membrane fusion. In this process the swimming force could also play a role, especially in the interaction and penetration of the spermatozoon through the ZP of the oocyte, and be involved in the fertilization failure. In the case of spermatozoa with altered motility, the analysis of the forces associated with the flagellar beating may provide further information for understanding anomalies related to sperm motility and their relationship to fertilization failure.

So far, few studies have reported on sperm cell mechanical properties. A work demonstrated that microcantilever can be used as nanomechanical sensor to study sperm motility [19]. In other works, sperm cells were most of the time anchored on surface, to simulate the anchoring of the sperm cell with ZP [20], or to measure the lashing forces by atomic force microscopy (AFM) [21], or lashing forces and torque by pipette-based approach [22]. To connect the value of these forces to the swimming and ZP penetration capability is not straightforward. The value of the force generated in a tail bending alone does not consider all the components involved in the generation of a swimming thrust. It is instead essential to take into account the number of beating per unit of time, the difference weight of slow and fast motions, as well as both linear and circular motions, the latter being lost when the spermatozoon is kept flat on a surface.

The implementation of novel approaches is thus important to have a complete understanding of the spermatozoa beating forces in conditions closer to the physiological ones. In this sense the Fluidic Force Microscopy (FluidFM), combining the Atomic Force Microscopy with a microfluidic cantilever, represents a powerful and versatile technique for studying single cell mechanics that allows to analyze and measure cell adhesion forces [23–26], and spermatozoa flagellar beating forces and frequencies, in a freely floating condition [27].

In this work we used FluidFM to study the flagellar beating forces of human spermatozoa. The forces are quantified for spermatozoa from semen sample with different degree of progressive motility. Our findings highlight that the spermatozoa from sperm samples with varying degrees of progressive motility develop significantly different flagellar forces. Spermatozoa with linear movement, in particular, exhibit higher forces than those with circular movement, indicating that beside the unpaired directionality, which slow-down the navigation through the female reproductive tract, those sperm cells may also experience a less effective sperm-oocyte interaction. Overall, there is still much to learn about the mechanics of flagellar movement and the factors that contribute to the generation of these forces. Understanding the forces exerted by sperm cells is important for understanding the functionality of these cells, the mechanisms of fertilization and for the development of treatments for fertility issues.

## Materials and methods

### Collection and preparation of human spermatozoa samples

**Fig. 1:**
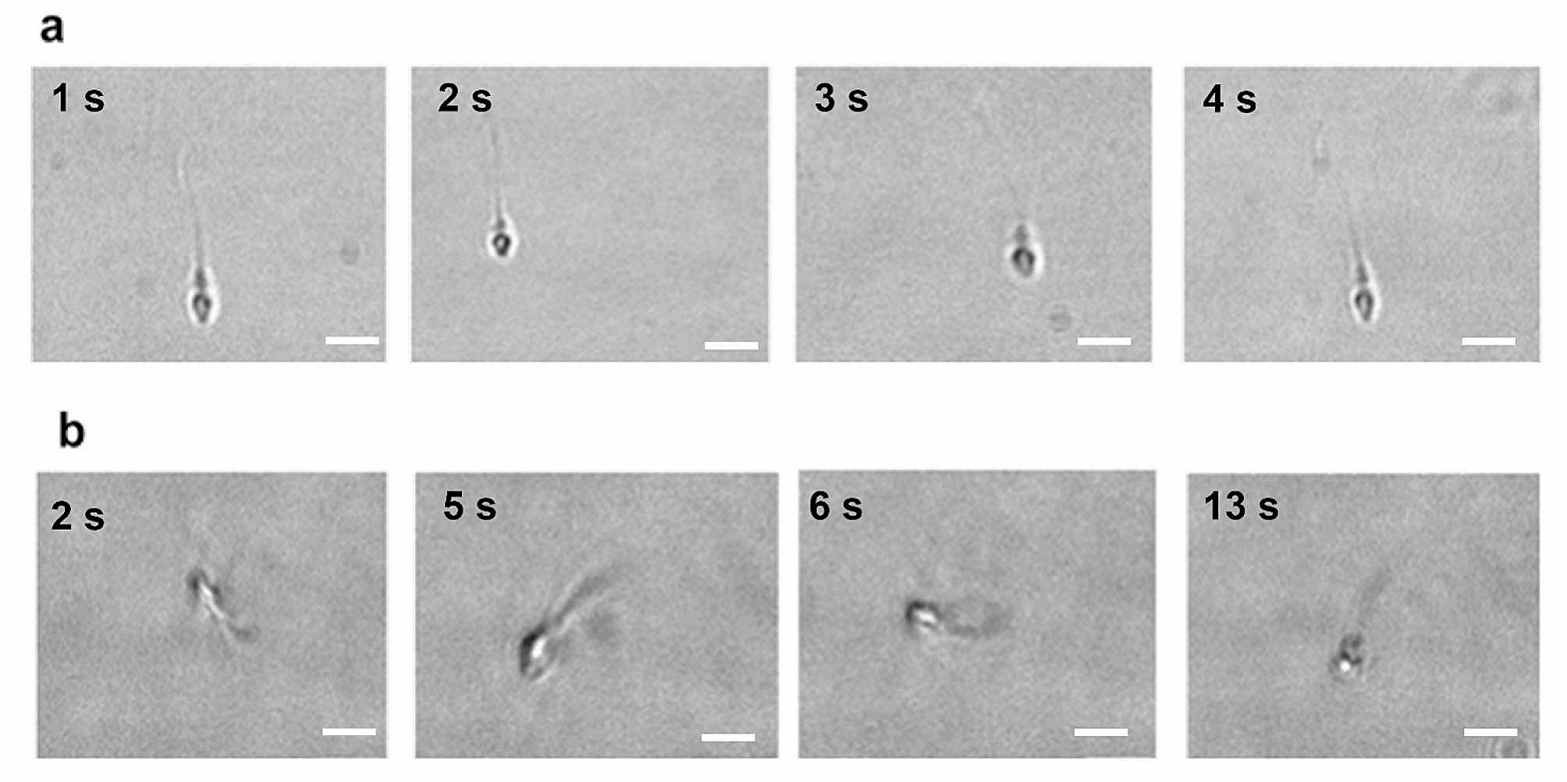
A sequence of frames extracted from movies acquired for sperm cells having two different motile behaviors: linear trajectory **(a)** and circular movement **(b)**. Scale bar: 5 μm

Semen samples were obtained from 12 men undergoing routine infertility investigations at Assisted Reproduction Unit of the Institute for Maternal and Child Health IRCCS Burlo Garofolo, Trieste, Italy, with written informed consent. All studies and procedures have been performed in accordance with ethical standards of Helsinki and approved by Regional Ethical committee (CEUR-2019-Os-19; Prot. N.0035571/P/GEN/ARCS, 05/12/2019). After capacitation by swim-up procedure, the spermatozoa of different semen samples were evaluated for their motility characteristics (see Supporting Information). On the base of this evaluation, semen samples were divided in two groups: the first group (5 semen samples from 5 different men) where all spermatozoa swim with a linear trajectory (100% progressive motility) (Fig. 1a), and a second one (from 7 semen samples from 7 different men) with a reduced progressive motility (80 − 20% progressive motility) where two motility behaviors were mainly present: spermatozoa with linear motility and others that swim with a circular movement (Fig. 1b). Images were captured at a camera frequency of 1 Hz for linear progressive spermatozoa, and 5 Hz for spinning top spermatozoa to better visualize their rotating behavior.

### FluidFM measurements of human spermatozoa

For FluidFM experiments, the spermatozoa are diluted with ASPTM (Vitrolife), a modified protein free HTF medium buffered with bicarbonate and HEPES and containing heparin and gentamicin, to a concentration of 30.000 sperm cells/ml. A single human sperm cell is trapped by using a FluidFM probe, an AFM cantilever with a nominal spring constant of 0.3 N/m which integrates a micropipette with a 2 μm aperture (Cytosurge, Zurich) mounted on a JPK Nanowizard II AFM (JPK instruments, Berlin). The conversion of the cantilever deflection into force (nN) is made as in standard AFM cantilever.

The spring constant of the FluidFM probes is calibrated using the thermal noise method prior to each measurement [28]. The bending of the cantilever is measured by optical beam deflection, and the position of the beam on a photodetector (V) is recorded. Before each experiment, the deflection sensitivity (S) of the cantilever is measured and the force (F) is derived from the following equation:

F (nN) = V (volts) S (nm/volts) K (nN/nm).

Because of the cantilever geometry and the AFM-like detection system, the deflections and thus the recorded forces should be intended always as limited to their z- component, or better the z-component of the thrust resulting from the spermatozoon beating. However, since spermatozoa have been captured in all orientations, and even during the individual experiments they may have changed the orientation, we can reasonably assume that the maximum thrust recorded for each experimental condition is a good approximation of the overall thrust, or at least set a minimum limit for it. With this warning in mind, in the following, we will refer to our measurement with the more general indication of “beating forces”, without loss of generality of the proposed argumentations.

**Fig. 2:**
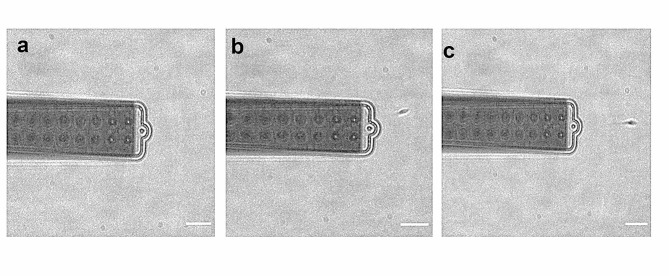
A sequence of frames extracted from a movie of a sperm cell being captured and released by FluidFM probe. When cantilever is far away from the sperm cell no pressure is applied **(a)**, the sperm cell is captured by a negative pressure, which is then reduced for force measurement of the motile sperm cell **(b)**, positive pressure is applied for sperm cell release **(c)**. Scale bar 10 μm

In Fig. 2, a sequence of images illustrates the trapping of a single sperm cell. In the trapping procedure, the cantilever is moved close or on the top of the spermatozoon without applying any pressure (Fig. 2a), when the cantilever is close enough to the sperm cell a negative pressure is applied to catch it. In the case of spermatozoa with circular movement, a pressure of -800 mBar is needed to trap most of the sperm cell, while a pressure of -200 mBar is enough to trap spermatozoa with linear motility. Once the sperm cell is trapped, the pressure is decreased to perform measurement (Fig. 2b). In case of spermatozoa with circular motility the pressure is lowered to -200 mBar and to -20 mBar in case of spermatozoa with linear motility. Several reasons may be at the origin of this difference in the pressure used to capture and keep the two group of cells. The more obvious would be a difference in the swimming thrust, but as we will show in the results, circular motility sperms exert lower forces than linear motility sperms. Other possible explanations include different hydrodynamics field typical of the two motions that makes the suction force more or less effective, a different size of the two kinds of cells, which although not easily appreciated at the optical microscope can matter with respect to the 2 μm-wide aperture, or a more shaking motion of the circular motile sperms, that is less effective as a thrust, but more effective in the detaching. However, since a detailed investigation of this effect is not relevant to the understanding of the fertilization process, we did not investigate it further. Therefore, we always used the lowest pressure that allows us to catch and keep the spermatozoa attached to the probe. The deflection of the cantilever associated with the sperm motion is recorded for an interval time of two minutes at a sampling rate of 1000 Hz. The resulting cantilever deflections are converted into force by using the sensitivity value and spring constant obtained in the calibration procedure. To monitor the status of the sperm cell while measuring, the trace is recorded simultaneously with a time lapse movie acquired by an XM10 monochrome CCD Camera (Olympus Corporation, Tokyo, Japan) and 40X objective lens. The traces for the spermatozoa that ceased to move while being measured were eliminated from the study, since the lack of flagellar movement made a strong indication that the sperm cell was in distress. The sperm cell can be released following the measurement by applying positive pressure of 1000 mBar (Fig. 2c), and in the majority of cases, it is still motile (Movie S1). After each measurement, the FluidFM probe is cleaned by immersions in a warm bleach 5% solution and then in a warm milliQ solution, in both cases applying cyclic steps of positive and a negative pressure to eliminate cell debris from the probe. Meanwhile the petri-dish containing the sperm cells is transferred into the incubator to avoid sperm cell suffering.

The measurements were performed on *n* = 15 sperm cells for semen sample with 100% progressive motility, all trapped by the tail, while in semen sample with progressive motility from 80 to 20% we identified and measured spermatozoa swimming linearly that were trapped by the tail (*n* = 16), or by the head/midpiece (*n* = 10) and sperm cell with circular motility (*n* = 17).

### Analysis of the force-time deflection traces

To isolate the frequencies of the beating flagellum and obtain the forces associated with these frequencies, the deflection traces are processed by Fast Fourier transform (FFT) and subsequently by Inverse Fast Fourier transform (IFFT) by using Igor Pro 6.37. Specifically, different frequency ranges were isolated from the FFT plot taking into account both the imaginary and the real part of the data. Then the FFT plots were inverse transformed to come back to the real space where the contribution of the frequencies to the original deflection signal can be isolated. Then an IFFT of the selected ranges is performed and the contribution of the single frequency ranges to the force signal is obtained. The direction of the sperm cell motion during the measurement cannot be controlled and this affects the amplitude of the force signal, as result for each sperm cell the maximum value of the force in recording interval was considered and evaluated as the relevant parameter. Moreover, the residual negative pressure applied to hold the sperm cell, although reduced during measurements can contribute to the bending of the cantilever. However, since the force measurements are dynamic and differential, that bending represents a baseline which is automatically subtracted by the data analysis process. The movies associated with deflection traces were processed by ImageJ free software.

### Statistical analysis

Statistical analysis was performed using GraphPad Prism (version 8.4.3). Statistical significance was evaluated by one-way ANOVA (Krusall Wallis test, Dunns comparison) *p* ≤ 0.05 (*), *p* ≤ 0.01 (**), *p* ≤ 0.001 (***) were considered significant.

## Results and discussions

### The beating forces of human spermatozoa

In general, the human sperm samples are characterized by a high level of heterogeneity, but when examined at the optical microscope, two main motile behaviors can be identified: the linear and the circular one. Semen samples with less than 40% total motile or 32% progressively motile spermatozoa are considered to be asthenozoospermic, a condition characterized by disorders in sperm motility correlated with the men infertility [29]. The knowledge of the features and function of spermatozoa motility are essential to better understand the causes of their functional alterations in men infertility. The advent of novel technological approaches enabled to evaluate and characterize features unexplored up to now. Among them, the FluidFM, whose microfluidic cantilever enables to trap and release a single cell can be exploited to evaluate the force exerted by single sperm cell, as we have demonstrated in our previous work [27].

**Fig. 3:**
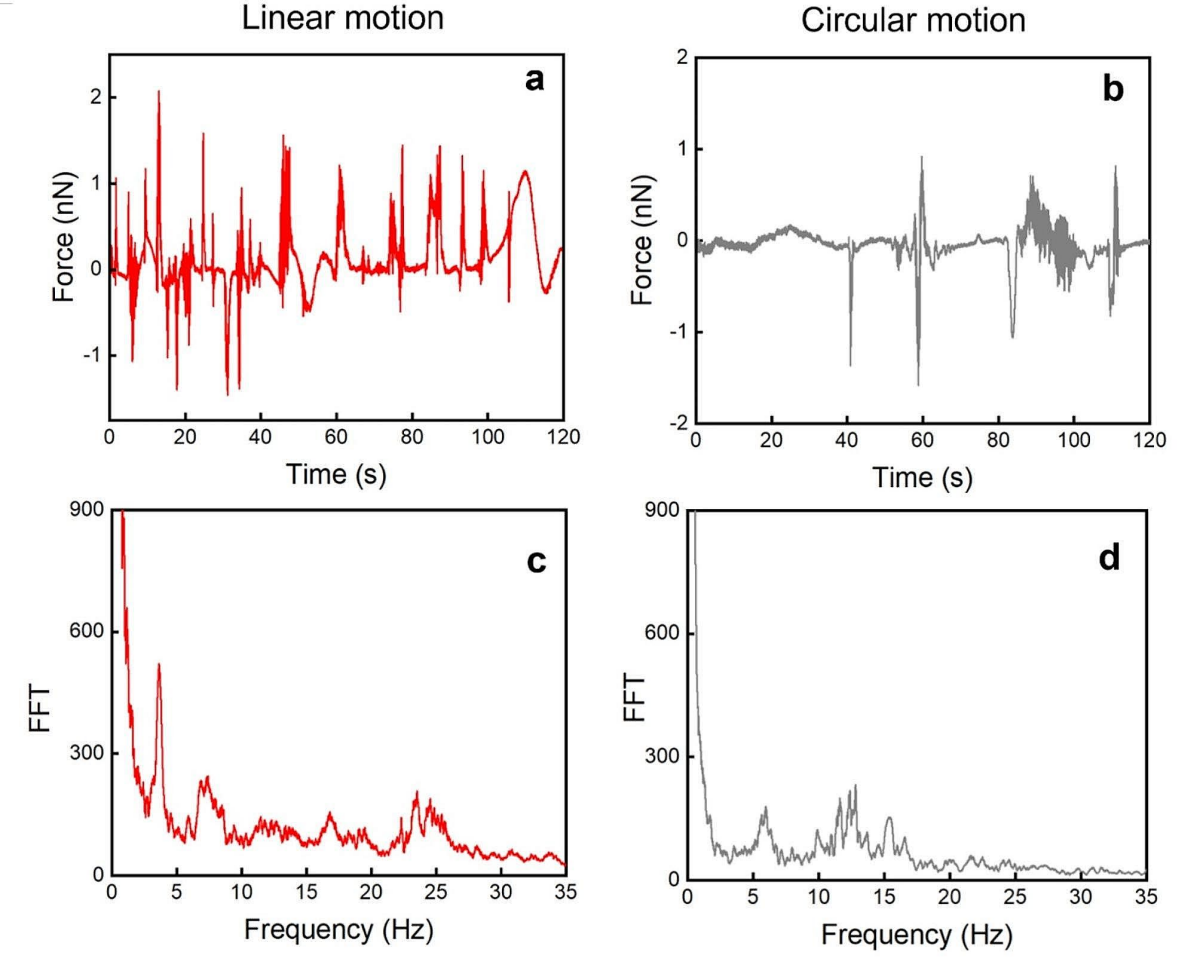
Representative cantilever oscillations **(a, b)** and Fast Fourier transform **(c, d)** of the signal when a spermatozoon with progressive linear motility is linked to the cantilever **(a, c)** or when one with circular movement **(b, c)** is trapped

Here we focus on the investigation and quantification of the beating forces associated with human spermatozoa having different motile behavior. Once the sperm cell is captured by the FluidFM cantilever, its deflection caused by cell movements is measured as the cantilever deflections vs. time. In Fig. 3(a,b) examples of the traces obtained for spermatozoa with linear and circular motility are shown respectively. The trace obtained for a linearly progressive spermatozoon shows numerous and frequent oscillations for the entire duration of the measurement (Fig. 3a), while in the case of circular motility, the trace shows a lower number of oscillations distant in time (Fig. 3b). The positive and negative sign of forces along the y axis depends on the different swimming directions and trajectory of the spermatozoon when it is moving on the cantilever. A closer look of these traces highlights the presence of pattern with periodic oscillations. The different frequencies were isolated by a FFT of the force-time plot. FFT magnitude of the oscillation traces for a linearly progressive spermatozoon and a circular one is reported in Fig. 3(c) and Fig. 3(d), respectively. The range of frequencies characteristic of sperm cells depends on the animal species and image-based approaches and video microscopy, has already described that in human spermatozoa the relevant range of beating frequencies is 0–30 Hz [5, 30]. In accordance with the data present in literature, we considered the ranges of frequencies from 0 up to 30 Hz and for a constant interval of 3 Hz. As result the frequency range considered are: 0–2 Hz, 3–5 Hz, 6–8 Hz, 9–11 Hz, 12–14 Hz, 15–17 Hz, 18–20 Hz, 21–23 Hz, 24–26 Hz and 27–29 Hz. The interval width of 3 Hz is the smallest frequency that allows to include an integer number of peaks for each interval.

**Fig. 4:**
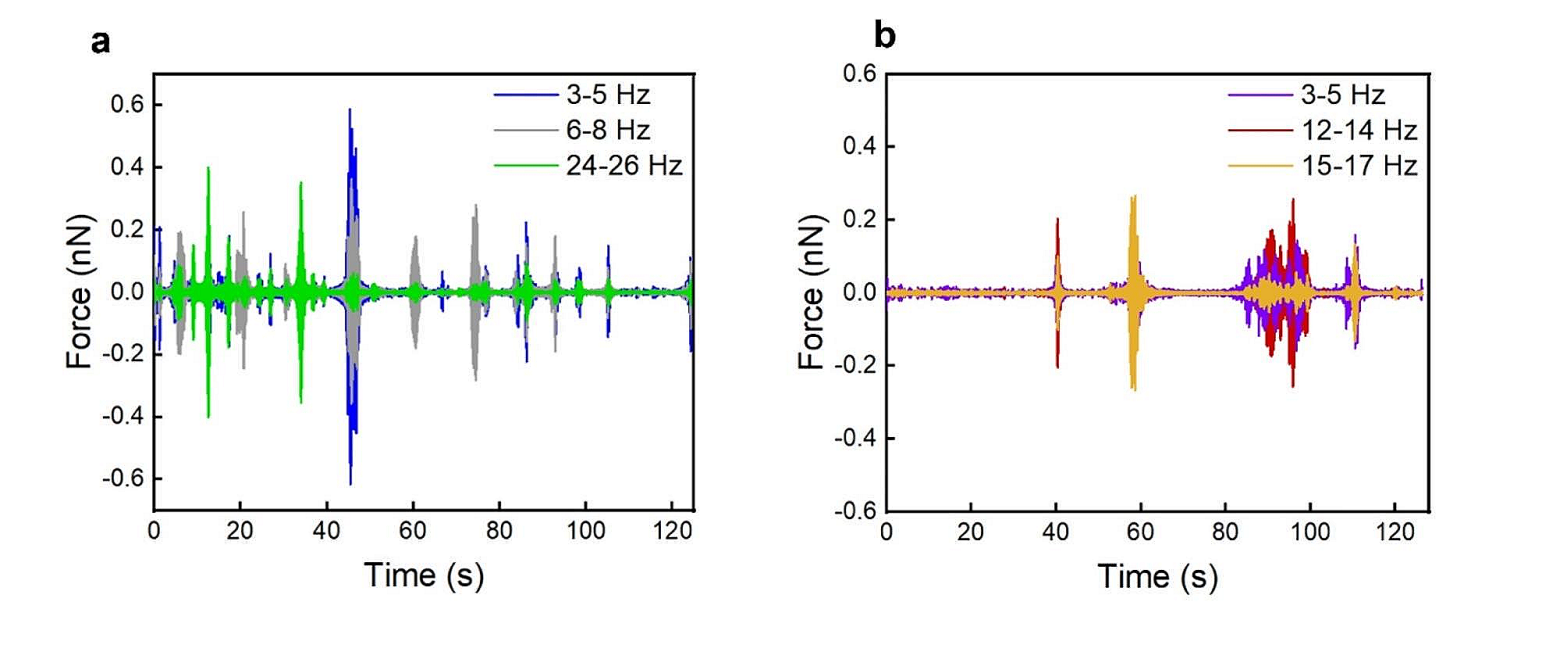
Inverse Fast Fourier transform of the main frequency ranges of the flagellar beating for a linear spermatozoon **(a)** and a circular one **(b)**

To obtain the forces associated with the frequencies detected an IFFT is performed. Figure 4(a) shows the IFFT of three ranges of frequencies (3–5 Hz, 6–8 Hz and 24–26 Hz) for a spermatozoon with linear motility, while Fig. 4(b) displays the ranges of 3–5 Hz, 12–14 Hz and 15–17 Hz for a spermatozoon with circular motility. We can observe that the amplitude of each frequency changes independently during the recording time and every frequency reaches a maximum at different times. In addition, the swimming direction of the sperm cell trapped on the tip of the cantilever alters the detected intensity of the force: the maximum force is measured when it moves perpendicular to the cantilever plane because this orientation of movement has the greatest impact on the vertical deflection of the cantilever, while a lower force is measured when the spermatozoon moves in the same plane of the cantilever [27]. For these reasons, from the IFFT graph the maximum force values are considered to evaluate the flagellar beating forces of the spermatozoa with different motile behaviors.

### The flagellar beating forces do not change as respect to the trapping region of spermatozoa

**Fig. 5:**
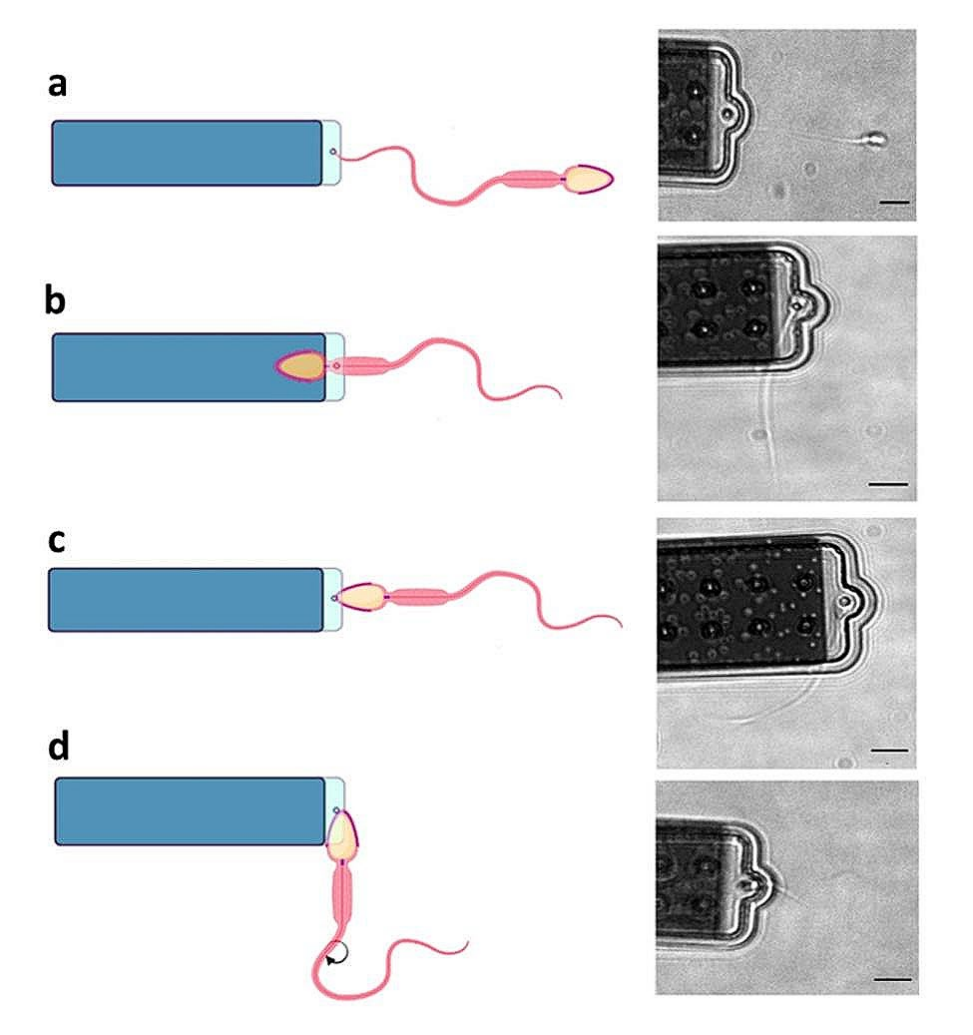
A sketch of the sperm cell trapping regions, as well as representative bright field images of sperm cells trapped by the various cellular regions: tail **(a)**, midpiece **(b)** head **(c)**, and head **(d)**. Scale bar 5 μm

A sperm cell has a peculiar elongated shape, optimized for their function, that includes head, midpiece and tail, and for this reason the trapping region can vary as shown in Fig. 5. According to sperm sample, we have trapped spermatozoa differently and investigated whether the trapping region could affect the movement transduction to the cantilever and thus the force intensity measured. In semen sample with 100% progressive motility, only spermatozoa trapping by the tail was successful (Fig. 5a). On the other hand, from semen sample with 80%-20% progressive motility spermatozoa with linear motility were trapped both by the head/midpiece and by the tail (Fig. 5b, c). Finally, spermatozoa with circular motility were captured only by the head, owing to their swimming behavior (Fig. 5d). In order to understand whether the trapping region could influence the force exerted by the cell we compared the maximum force of spermatozoa with linear motility when trapped by the tail, head or midpiece.

**Fig. 6:**
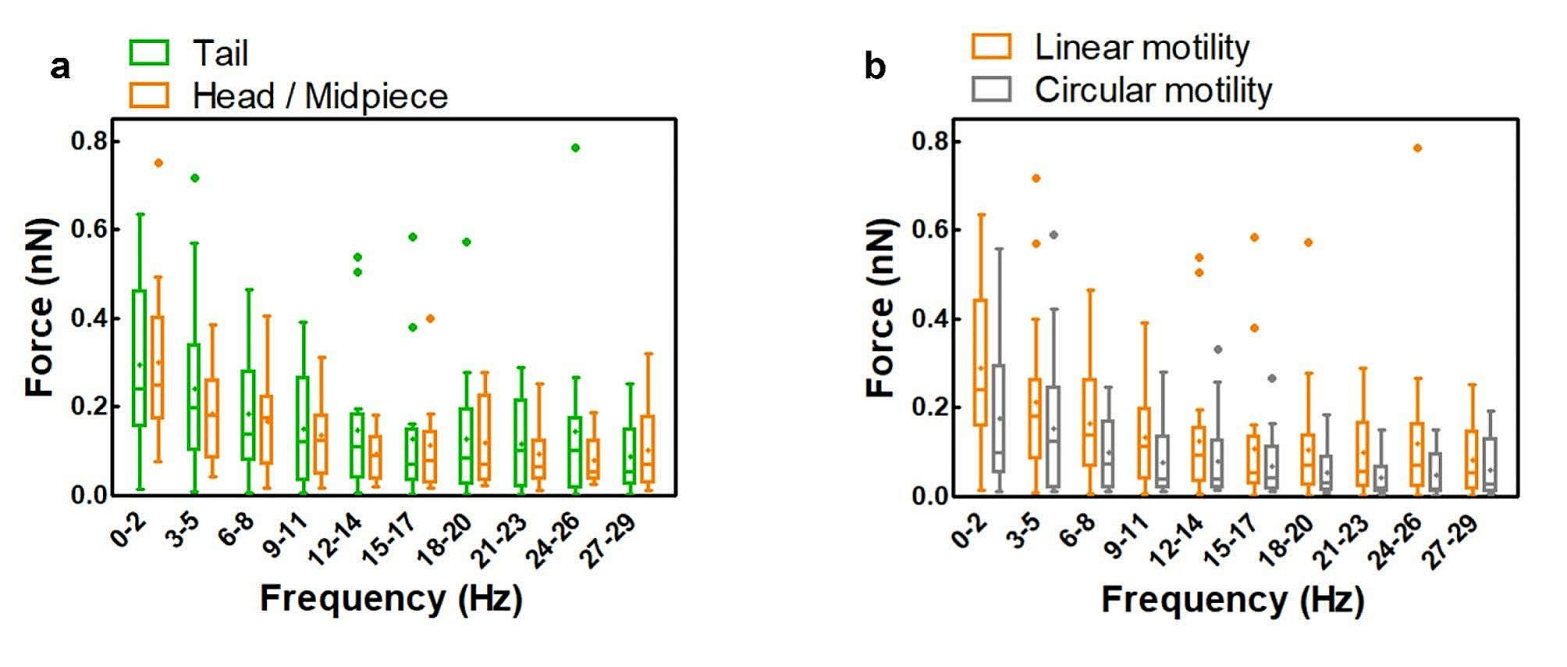
Semen samples with progressive motility from 80–20%: values of forces exerted by spermatozoa trapped by the tail (*n* = 16) and by the head/midpiece (*n* = 10) **(a)**, and comparison of forces exerted by spermatozoa with linear (*n* = 26) and circular motility (*n* = 17) **(b)**. Data are reported in box plots (+) shows the mean, while (-) indicates the median value

In this case we observed that, for each range of frequencies, there are no differences between the maximum forces of the spermatozoa trapped from different regions (Fig. 6a). This result indicates that, regardless of the cellular region where the human spermatozoon is captured, the detected flagellar beating forces do not significantly change. Furthermore, in semen sample with 80%-20% progressive motility, we compared the forces associated with spermatozoa having the two different motility behaviors (Fig. 6b). For linear motile spermatozoa we grouped the data from those trapped by the head/midpiece and tail since no significant differences were previously observed between the two trapping modalities. Although, between the linear and circular motility, no significant difference in the maximum forces for each range of frequencies were observed, a general decrease of maximum force values for the circular motile spermatozoa, as compared to the linear ones, can be noticed, especially at lower frequencies (0–2 Hz).

### The beating forces of spermatozoa from 100% progressive semen sample are greater than those of spermatozoa from 20 to 80% progressive semen sample

**Fig. 7:**
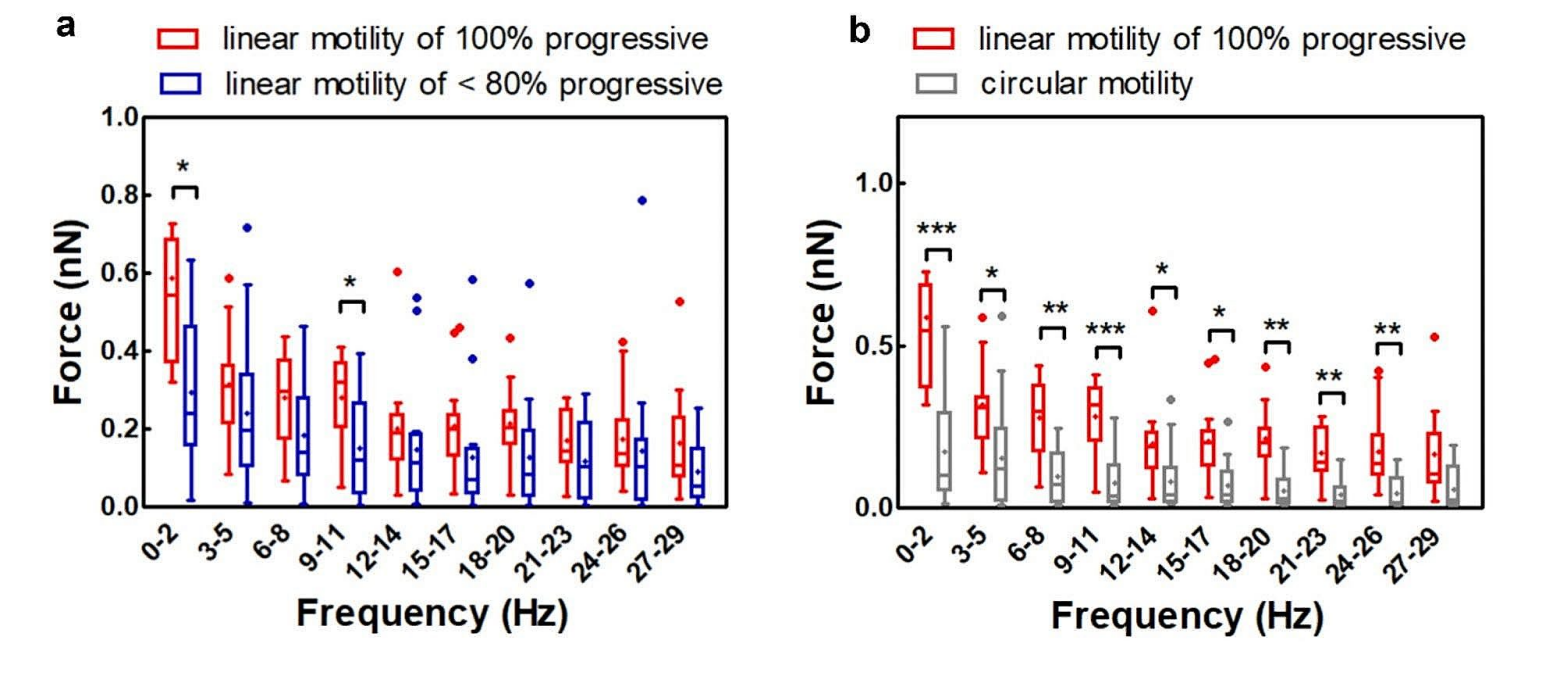
Comparison of the forces exerted by the spermatozoa with linear motility from in semen sample with 100% progressive motility (*n* = 15) and from semen sample with a progressive motility lower than 80% (*n* = 16), in both cases, the spermatozoa are trapped by the tail **(a)**. Forces of spermatozoa swimming linearly in samples with 100% progressive motility (*n* = 15) and spermatozoa with circular movement (*n* = 17), the circular motile sperm cells are trapped by the head, while the linear motile spermatozoa by the tail **(b)**. Data are reported in box plots (+) shows the mean, while (-) indicates the median value. Significance level *p ≤ 0.05, **p ≤ 0.01, and ***p ≤ 0.001

To evaluate whether the beating forces of spermatozoa change in semen sample with different degree of progressive motility, we compared the flagellar beating forces of human spermatozoa with 100% of linear motile spermatozoa with those 80%-20% progressive motility. Figure 7a shows the forces associated with sperm cells that move linearly both in 100% and 20–80% progressive sample, all trapped by the tail. We can see a general decrease in forces exerted by linear motile spermatozoa in 80%-20% of the samples when compared to 100% progressive samples, with significant differences in the frequency ranges of 0–2 Hz and 9–11 Hz. In the hyperactivated sperm cells lower frequencies of the flagellar beating are those supposed to be important both for the propagation of the spermatozoa in the female reproductive tract and for cell sperm penetration through the ZP [20, 31]. The ZP can have plastic or elastic characteristics depending on the deformation. It has been observed that beating at lower frequencies are associated with the deformation of ZP that allows a sperm cell to fuse with the oocyte [20]. Taking into account that spermatozoa swimming linearly, from samples with 80 − 20% progressive motility, exhibit lower forces associated with low frequencies (0-2 Hz and 9–11 Hz), it is likely that these spermatozoa will not be able to fully exploit the plastic behavior of the ZP. These lower forces, on the other hand, may be also associated with the inability of these sperm cells to penetrate the ZP leading to a failure of acrosome reaction. Some authors proposed indeed that the passage of motile sperm through the ZP triggers the acrosome reaction and that a similar mechanism may be involved in reaction during physiological fertilization process [32]. Therefore, we can hypothesis that in semen samples with lower progressive motility, the spermatozoa moving in a linear progressive manner can reach the oocyte, but they may encounter some difficulty penetrating through the ZP, owing to lower thrust forces.

We then compared the forces exerted by spermatozoa with circular motility to linear motile spermatozoa from 100% progressive semen sample. The first is trapped on the FluidFM cantilever by the head, while the latter by the tail. Although the trapping regions of the analyzed human spermatozoa are different, we do not expect the data could affected by the trapping position as previously demonstrated for lower motility sperm cells. The distribution of maximum forces related to the different frequency ranges for the two groups of analyzed spermatozoa is shown in Fig. 7b. The forces exerted by linear motile sperm cells in 100% progressive semen samples are significantly higher than spermatozoa with circular motility for almost all ranges of frequencies.

The penetration of the human spermatozoon through the ZP is very likely a combined process involving a synergy of mechanical and biochemical events [33]. In comparison to the linear motile spermatozoa in the 100% progressive sample, the circular motile spermatozoa have a lower potential to reach the oocyte through the female reproductive tract, and probably also a lower ability to pierce the ZP. The combination of mechanical and biochemical events in the fertilization process can differ among the mammalian species. We discovered, indeed, that the flagellar beating forces exerted by mouse spermatozoa are around (3.77 ± 2.11) nN for low frequencies range (0–2 Hz) [27], which, for same frequencies, are more than 5 times higher than those obtained for human spermatozoa (0.6 ± 0.2) nN. This is in agreement also with the differences already described in the morphology, chromosome number, mitochondrial activity, structure of the flagellum and motility among the mammalian sperm species [34].

## Conclusion

We discovered that the forces exerted by human sperm cells are different according to the percentage of progressive motility of the semen sample as evaluated by clinical protocols. In particular linear motile spermatozoa of 100% progressive semen sample show significantly higher forces than circular motile spermatozoa. Healthy human spermatozoa must move with a force intensity that enable them to swim through the high viscous fluid in the female reproductive tract, while spermatozoa with low thrusting forces are slowed down in the female tract and cannot reach the oocyte. The low thrusting forces could lead to difficulties during different steps of the fertilization process. In particular, these forces can be associated with the failure of the movement of sperm cells from the cervix to the fallopian tube where the sperm-oocyte interaction occurs, but they could be also associated with the inability of sperm to penetrate the ZP. The etiology of 30–40% of cases of male infertility is unknown and these cases are called idiopathic [35]. The analysis of flagellar beating forces of human spermatozoa with different motility behavior and different speed could open to further clarifications about the causes of idiopathic male infertility.

### Electronic supplementary material

Below is the link to the electronic supplementary material.

Supplementary Material 1

Supplementary Material 2

## Data Availability

The data reported in the manuscript are available from the corresponding authors by request.
