# Supplementary material for: Flagellar beating forces of human spermatozoa with different motility behaviors
Source: Reprod Biol Endocrinol. 2024 Mar 6;22:28. doi: 10.1186/s12958-024-01197-8 (PMC10916019; doi:10.1186/s12958-024-01197-8)
Supplement: Supplementary file 2 — Supplementary Material 2 [file 12958_2024_1197_MOESM2_ESM.docx]

**Supporting Information**

**Flagellar beating forces of human spermatozoa with different motility behaviors**

Cristina Tufoni^1,2^†, Alice Battistella^2^†, Stefania Luppi^3^, Rita Boscolo^3^, Giuseppe Ricci^3,4^*, Marco Lazzarino^2^, Laura Andolfi^2^*.

*1 University of Trieste, 34100 Trieste, Italy*

*2 CNR-IOM SS 14 km 163.5 Area Science Park Basovizza 34149 Trieste Italy*

*3 Institute for Maternal and Child Health - IRCCS “Burlo Garofolo” – Trieste, Italy*

*4 Department of Medicine, Surgery and Health Sciences, University of Trieste, Trieste, Italy*

**1S. Semen treatments**

The sample is collected after a minimum of 2 days and a maximum of 7 days of sexual abstinence. It is obtained through masturbation and ejaculation into a clean container that has been confirmed to be non-toxic for spermatozoa. Immediately after ejaculation into the collection vessel, the ejaculate is typically a semi-solid coagulated mass or a gel-like clump, which begins to liquefy within a few minutes at room temperature, at which time a heterogeneous mixture of semi-solid lumps will be seen in the fluid. As liquefaction continues, the ejaculate becomes more homogeneous but still with a viscosity higher than water. A temperature of 37 °C will facilitate liquefaction. Then the spermatozoa are separated from seminal plasma and capacitated by pellet swim-up technique. This procedure allows sample enrichment of motile sperm cells and the elimination of cellular debris or non-viable cells. The pellet swim-up consists of diluting liquefied semen with an equal volume of HTF washing medium (Fujifilm, Irvine Scientific) containing 5% albumin (Origio MediCult, Denmark) and preheated to 37 °C. Then it is centrifuged at 400 x g for 10 minutes: the supernatant is removed and the pellet is stratified with 0, 5 ml of HTF medium with 5% albumin (Origio MediCult, Denmark) preheated to 37 °C. After an incubation of 30-45 minutes at 37 °C, the upper layer containing the capacitated spermatozoa is taken and analyzed (1).

**2S. Clinical evaluation of sperm motility**

The evaluation of capacitated sperm cells motility is carried out as reported by the Fifth edition of the WHO laboratory manual for the examination and processing of human semen (2). The procedure of motility assessment is performed at 37 °C with a heated microscope stage. An aliquot of capacitated sample after mixing is placed on a glass slide and covered with a coverslip. Then we waited for the sample of spermatozoa to stop drifting. At this point, the slide is observed with phase contrast optics 200x magnification. The spermatozoa were counted in an area at least 5 mm from the edge of the coverslip to avoid the effect of drying on motility and the sperm motility was evaluated by looking at all spermatozoa within a defined area of the field using an eyepiece reticle (Fig.1S).


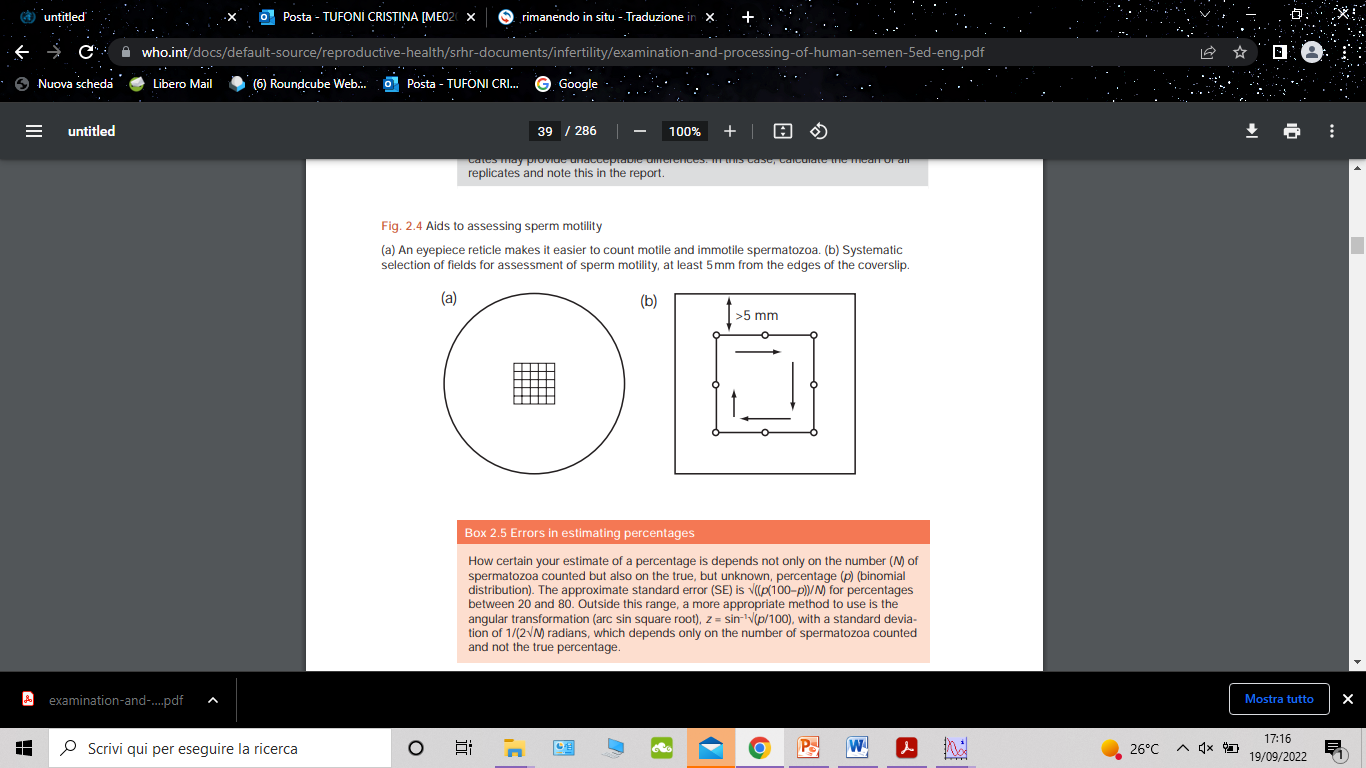


**Fig.1S**: (a) Eyepiece reticle used to count the different motile categories of spermatozoa; (b) selection of field for evaluation of sperm motility (2).

The eyepiece reticle allows evaluation of the same area of the slide during the analysis. The portion of the grid to be scanned depends on sperm concentration: in case of sample with few spermatozoa we evaluated the entire grid, while if the concentration of spermatozoa is high, we scored only the top row. The count was done quickly to avoid overestimating the number of motile spermatozoa considering both those present initially and those that swim into the grid during scoring. The spermatozoa motility was estimated in at least five fields in each replicate to obtain a percentage with an acceptably low sampling error. In this field of the grid, over 100 spermatozoa are considered to evaluate the percentage of the motile categories.

In this study, we considered: (i) semen samples with 100% progressive motility, which included all spermatozoa moving actively with a linear trajectory; and (ii) semen sample with progressive motility lower than 80%, which included different percentage of progressive spermatozoa while the rest being non-progressive spermatozoa (e.g., swimming in small circles or the flagellar force barely displacing the head).

**References**

(1) Ricci, G., Perticarari, S., Boscolo, R. et al. Leukocytospermia and sperm preparation - a flow cytometric study. Reprod Biol Endocrinol 7, 128 (2009).

(2) World Health Organization. WHO laboratory manual for the examination and processing of human semen. 5th ed. Geneva: World Health Organization (2010).
